# Spectrophotometric Methods for Measurement of Antioxidant Activity in Food and Pharmaceuticals

**DOI:** 10.3390/antiox11112213

**Published:** 2022-11-08

**Authors:** Marios C. Christodoulou, Jose C. Orellana Palacios, Golnaz Hesami, Shima Jafarzadeh, José M. Lorenzo, Rubén Domínguez, Andres Moreno, Milad Hadidi

**Affiliations:** 1 Department of Chemistry, University of Cyprus, Nicosia 1678, Cyprus; 2 Department of Organic Chemistry, Faculty of Chemical Sciences and Technologies, University of Castilla-La Mancha, 13071 Ciudad Real, Spain; 3 Department of Food Science and Technology, Sanandaj Branch, Islamic Azad University, Pasdaran St., Sanandaj P.O. Box 618, Iran; 4 School of Engineering, Edith Cowan University, Joondalup, WA 6027, Australia; 5 Centro Tecnológico de la Carne de Galicia, Avd. Galicia N° 4, Parque Tecnológico de Galicia, San Cibrao das Viñas, 32900 Ourense, Spain; 6 Área de Tecnología de los Alimentos, Facultad de Ciencias de Ourense, Universidade de Vigo, 32004 Ourense, Spain

**Keywords:** antioxidative activity, determination, free radicals, plant-based antioxidant, phenolic compounds, colorimetry

## Abstract

In recent years, there has been a growing interest in the application of antioxidants in food and pharmaceuticals due to their association with beneficial health effects against numerous oxidative-related human diseases. The antioxidant potential can be measured by various assays with specific mechanisms of action, including hydrogen atom transfer, single electron transfer, and targeted scavenging activities. Understanding the chemistry of mechanisms, advantages, and limitations of the methods is critical for the proper selection of techniques for the valid assessment of antioxidant activity in specific samples or conditions. There are various analytical techniques available for determining the antioxidant activity of biological samples, including food and plant extracts. The different methods are categorized into three main groups, such as spectrometry, chromatography, and electrochemistry techniques. Among these assays, spectrophotometric methods are considered the most common analytical technique for the determination of the antioxidant potential due to their sensitivity, rapidness, low cost, and reproducibility. This review covers the mechanism of actions and color changes that occur in each method. Furthermore, the advantages and limitations of spectrophotometric methods are described and discussed in this review.

## 1. Introduction

Many studies have been conducted related to the oxidation origin of free radicals and the general role of antioxidants since the beginning of the 21st century. This interest was generated because free radicals are highly reactive and unstable molecules with a significant impact on the human biological system even though they are neutral. In fact, some important lipid-derived compounds, such as aldehydes, can produce negative effects on human health, although these compounds can naturally form during food processing, mainly linked to thermal treatments [1]. Radicals’ significant activity is an outcome of an atom that carries an unpaired electron. Due to this lack of outer-shell electrons, they are constantly searching to bind with another atom or molecule to stabilize themselves [2]. Despite antioxidant defense mechanisms, human cell damage accelerates aging and can play a critical role in the development of other diseases [3,4]. Further oxidative modification of biological macromolecules (e.g., lipids, proteins, and DNA) can result in tissue injury [5]. In understanding these occurrences and preventing them, a higher quality of life may be gained.

Recently, extensive research has been classified into different types of free radicals. The three main categories are: reactive oxygen species (ROS), reactive nitrogen species (RNS), and reactive sulfur species (RSS), which are formed from oxygen, nitrogen, and sulfur atoms, respectively [6]. Examples of ROS, RNS, and RSS include hydrogen oxide, hydrogen peroxide, singlet oxygen, alko-xyradical, peroxyl-radical, nitrogen monoxide, nitric oxide superoxide anion, hydroxyl anions, alkyl-thiol, etc. [2,6,7]. More specifically, the ROS group includes lipid peroxidation products and protein carbonyl species while the RNS group includes nitric oxide and peroxynitrites. Nitric oxide plays a key role in DNA damage, inflammation, cancer cell growth, and apoptotic malfunction, even though it has a lifespan of only a fraction of a second. In addition, peroxynitrites have the potential to cause lipid peroxidation, DNA damage, and long-term damage to all biomolecules. Similarly, sulfur species (RSS) may act in unison to damage biomolecules and, hence, extensive damage to genes in DNA may result in genes that produce ineffective proteins [4,8]. The origin of radicals is not yet well defined, but our own body often produces free radicals in the process of breaking down nutrients to create the energy that allows our bodies to function. Endogenous sources are multifaction in mitochondria, peroxisomes, endoplasmic reticulum, phagocytic cells, etc. while some exogenous sources may be air pollution, ultraviolet radiation, alcohol, smoking, contact with heavy metals, pesticides, and certain drugs such as halothane and paracetamol [9].

Fortunately, antioxidants can neutralize free radicals and reduce the risk of damage [10]. Antioxidants have become rapidly known for their health-promoting capabilities. By definition, the term “antioxidant” refers to a class of compounds with synthetic or natural orientation, which can act as chain-breaking antioxidant inhibitors, stopping the chain reaction of free radicals by complexing with them [11]. Apart from stopping the formation mechanism, an antioxidant compound must be able to scavenge radicals and form new ones that are stable [12]. Natural compounds such as these can be found in fruits, roots, vegetables, and plants [13,14,15,16]. In addition to this, a study conducted by Yashin et al. [17] suggests that the most biologically active compounds are contained in various spices and herbs.

The main representatives of antioxidants are vitamins A, C, and E; beta-carotene; anthocyanidins; phenols; flavonoids; phenolic acids, etc. [17]. Certainly, natural antioxidants that are consumed daily in our diet can protect our bodies and act as anticarcinogenic agents. Higher antioxidant and anticancer activities are also demonstrated in cases where there is a synergetic effect between different antioxidant natural molecules [18,19,20]. Endogenous defenses in humans have gradually improved over time, resulting in a balance between free radicals and oxidative stress. Enzymatic antioxidants and non-enzymatic oxidants are the two main types of antioxidants found in humans [6]. Antioxidant defense mechanisms attempt to scavenge reactive oxygen species and prevent their formation, although they are not always successful. The antioxidant network is complex, containing substances that are both endogenous and ingested. Enzymes called superoxide dismutase (SOD) convert O_2_ to H_2_O_2_ and eliminate it from the body [21]. However, because of the blood–brain barrier, antioxidants sometimes fail to provide adequate protection [22]. Numerous studies have demonstrated the necessity of antioxidants, but currently, the preferred choice of determination method is a controversial challenge. Antibody, fluorescence, light emission, spectrophotometric, chromatography, and electrophoretic techniques are the most widely used quantification procedures for determining the total antioxidant activity (TAA) [6,23,24]. Although spectrophotometric methods are standard and very basic techniques, they have been used due to their simplicity and low cost. Established spectrophotometric methods such as ABTS, DPPH, FC, FRAP, and CUPRAC have been used for years, as they are characterized by rapidity, reliability, and simplicity [25]. Understanding the basic principles is critical for selecting the best suitable procedure [5,26,27]. The main goal of this review is to classify, outline, and discuss each of these spectrophotometric assays along with their mechanism of action in order to present to the readers the advantages and disadvantages while providing a thorough understanding of free radicals and natural antioxidant molecules.

## 2. Oxidation Process and Radicals

As mentioned before, free radicals are chemical entities (atoms, molecules, or ions), that have a single unpaired electron in one of their outer orbits, which makes them unstable and reactive [28]. The outcome is the formation of more radicals or unwanted side products as an electron attempts to bind with another. The majority have a half-life that depends on their environment. For example, the half-life of NO• or most oxygen species is a few minutes whereas the half-life of sulfur anion free radical is seconds. The initiation of the generation of radicals is any source of heat, ultraviolet irradiation, and air pollution, or naturally occurs in mitochondria. In humans, small molecules, peptides, proteins, and enzymes mostly contain nitrogen, oxygen, and sulfur, which serve a variety of functions in living creatures [29]. Nitrogen and oxygen are usually bonded in ‘chains’ of two to three atoms (peroxides, ozone, dinitrogen trioxide, etc.) while sulfur chains can be considerably longer (Table 1). These atoms have many oxidation states, and sometimes, during certain events and under certain circumstances, they release free radicals as side products [2,28,29]. During the propagation step, free radicals react with other molecules until their termination, where the free radicals bind together in a way that the chain is no longer propagated.

Reactive oxygen (ROS), nitrogen (RNS), and sulfur (RSS) species are formed both endogenously and exogenously [2]. The most prevalent free radicals that cause harm to biological systems are oxygen-free radicals, also known as ROS [30]. They are produced as a by-product of biochemical reactions by neutrophils and macrophages in mitochondria, peroxisomes, and other organelles [31]. Activated forms of ROS are illustrated in Table 1 and are usually separated as small particles that do not contain carbon atoms, such as •OH, in contrast with forms such as ROO•. Reactive sulfur species have also received attention for their role in oxidative stress, a phenomenon caused by an imbalance between the production and accumulation of reactive species in cells and tissues and the ability of a biological system to detoxify these reactive products [32]. Normally, sulfur radicals can be produced by hydrogen donation, enzymatic oxidation, and interaction with reactive oxygen species such as hydrogen peroxide, singlet oxygen, peroxynitrite, and superoxide [29]. When cellular thiols are oxidized, they form species that can oxidize and inhibit the action of proteins and enzymes. Currently, known sulfur-free radicals include disulfides and monosulifes, disulfide-S-oxides, and sulfenic acid agents (Table 1). According to Abedinzadeh et al. [33], the formation of highly reactive sulfur radicals participates in several different reactions, which lead to disulfide radical anion, thiyl peroxyl radical, and others. On the other hand, reactive nitrogen species (RNS) are a class of antimicrobial compounds produced by nitric oxide and superoxide. When they combine, they are converted to a peroxynitrite free radical. Rapid protonation of peroxynitrite anion in vivo gives peroxynitrous acid (ONOOH), which acts as an electrophilic nitrating agent for tyrosine and tryptophan sidechains in proteins. The decomposition of peroxynitrous acid can generate hydroxyl radicals, which can subsequently damage human DNA [34]. Further damage to DNA clones has been reported as the presence of nitric oxide free radical is related to dose-dependent DNA strand breaks and the transformation from cytosine to uracil and 5-methylcytosine to thymine [35]. The endogenous antioxidant defense system can also be overwhelmed by ROS, RNS, and RSS, resulting in cellular damage and dysfunction, which leads to a variety of illnesses. ROS and RNS are key regulatory mediators in signaling pathways at low concentrations, but they are toxic in moderate and high quantities, inactivating critical cellular components [36].

Nitric oxide (NO•) has two purposes in health and sickness and its level influences both. Nitric oxide has the potential to act as an active marker of cancer progression during physiological and pathological processes by encouraging angiogenesis or the production of new blood vessels [37]. Furthermore, by upregulating p53, poly (ADP-ribose) polymerase, and DNA-dependent protein kinase, NO• may affect tumor DNA repair processes (DNA-PK). The use of NO• in cancer research has significant therapeutic implications for disease detection and treatment. At the same time, the ratio of ROS/RNS is engaged in a range of physiological activities, including immunological function (i.e., protection against harmful microorganisms), cellular signaling pathways, mitogenic response, and redox regulation, and has beneficial effects at moderate or low levels. However, at higher ratios of ROS/RNS, oxidative and nitrosative stress can occur, which can destroy biomolecules as the antioxidant and oxidant levels are unbalanced [9,38]. Increasingly more free radicals build up, causing extensive damage to macromolecules, including nucleic acids, proteins, and lipids.

Peroxidation of lipid products and protein carbonyls is only one of the side effects of ROS when nitric oxide and peroxynitrites are produced from nitrogen radicals [4]. Therefore, any anomalies that occur in these crucial structural components have been related to the onset of a variety of neurodegenerative diseases, such as Alzheimer’s, Parkinson’s, etc. [39]. A study by Porter et al. [40] indicated that malondialdehyde (MDA, ROS agent) interacts with low-density lipoproteins and, as a result, lipid peroxidation forms, which indirectly causes atherosclerosis. Additionally, ONOO^−^ is another major RNS player that acts as a lipid peroxidation catalyst, which causes membrane and lipoprotein disruption. In the growth of cancer, ONOO^−^ and MDA act as cytotoxic and mutagenic agents, promoting DNA damage through mutations, resulting in decreased tumor suppressor gene expression or enhanced oncogene expression [41]. The possible consequences of the effects of lipids and proteins include tissue damage, neurological illnesses, cancer, cardiovascular diseases, cataracts, rheumatoid arthritis, asthma, stroke, myocardial infarction, chronic heart failure, diabetes, and many other neurodegenerative disorders [4,22,34].

## 3. Classification of Natural Antioxidants

Antioxidants can be separated into two main categories: synthetic and natural, which are derivatives of fruits, herbs, and plants [42,43,44]. Additionally, different plant by-products are also an economic source of natural antioxidants [45]. Nowadays, the employment of synthetic antioxidants in food, such as butylated hydroxytoluene (BHT) or butylated hydroxyanisole (BHA), has raised social concern, as these compounds are effective and relatively cheap to produce, but they can generate allergies and serious problems for human health in the long term [46,47]. This is also the major reason why many companies are trying to replace compounds synthesized in the laboratory with natural antioxidants to prevent oxidation in food products and secure a healthier lifestyle [48]. However, antioxidants must meet some of the following criteria to be utilized in this task. Firstly, they must be efficient, and their utilization must be cost-effective. Secondly, large-scale usage is not practicable if they are too expensive or complex to synthesize or isolate. Finally, they need to be kept at low concentrations, as these substances can be harmful to people at extremely high levels.

**Figure 1:**
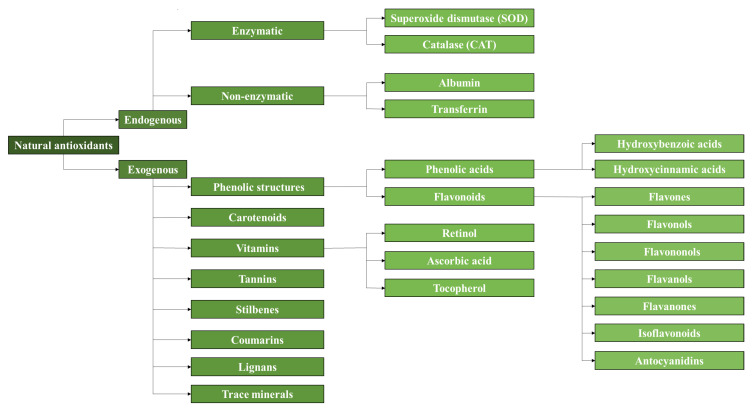
Classification of natural antioxidants [46].

It is well known that plants, fruits, vegetables, herbs, seeds, and other natural sources present a large cocktail of antioxidants, such as phenolic compounds, carotenoids, and vitamins [16]. Due to this fact, a diet rich in fruits and vegetables is usually recommended in order to receive all the benefits. By doing so, it is possible to prevent or delay certain diseases, such as cardiovascular diseases or diabetes [46,49]. This variety of phenolic acids and flavonoids, along with a general classification of antioxidants, is shown in Figure 1. Additionally, some by-products of the food and agricultural industries, such as shells or peels, have been and continue to be investigated for the extraction of antioxidants. It is also important to note that, depending on the type of the plant and its morphological parts, the antioxidant capacity can vary significantly, as the antioxidant capacity of the leaves is not the same as that of the stem [27]. Based on this, different processes such as extraction, separation, and characterization of natural antioxidants have been investigated over the years.

According to Figure 1, natural antioxidants can be classified into endogenous and exogenous antioxidants. Endogenous antioxidants are synthesized internally by the metabolism while exogenous antioxidants are obtained mostly in plants. The combination of endogenous and exogenous antioxidants in the human body helps maintain the nucleophilic tone, which translates into a healthy physical state [49]. Endogenous antioxidants can be divided into enzymatic and non-enzymatic. Enzymatic antioxidants act as the first line of defense in the human body while non-enzymatic antioxidants usually act as the second line of defense. The main representatives of enzymatic antioxidants with the highest effectiveness are superoxide dismutase (SOD) and catalase (CAT). SOD is responsible for obtaining O_2_ and H_2_O_2_ from the O_2_^−^ radical. Then, CAT takes H_2_O_2_ and converts it to H_2_O and O_2_ [26]. Non-enzymatic proteins such as albumin and transferrin are also endogenous antioxidants. Proteins of this type are capable of trapping metal ions, avoiding the formation of new reactive species [49]. Exogenous antioxidants can also be divided into many different compounds. The most significant ones that are present in the diet of an average person are phenolic compounds such as flavonoids and phenolic acids, vitamins such as ascorbic acid (C) and tocopherol (E), and carotenoids [47].

In the case of vitamins, those that are water-soluble, such as ascorbic acid, are responsible for stopping free radicals present in the aqueous phase. Fat-soluble vitamins, such as tocopherol, are present in cell membranes, preventing their degradation [26]. The largest groups of exogenous natural antioxidants are phenolic structures. Within them, a distinction can be made between phenolic acids and flavonoids. Both are present in plants and this is also the reason why plant-derived foods contain a large amount of these exogenous antioxidants. As can be seen in Figure 1, these acids are divided into two groups depending on whether they are formed from benzoic or cinnamic acid. Some examples are caffeic acid (derived from hydroxycinnamic acid) and vanillic acid (derived from hydroxybenzoic acid). It should be noted that compounds derived from hydroxybenzoic acid have a lower antioxidant capacity than those derived from hydroxycinnamic acid. In general, it is estimated that the average person consumes about 200 mg/day of phenolic acid during the day [46].

On the other hand, flavonoids are present in large amounts in plants, formed from primary metabolites. Plants can transform the amino acids tyrosine and phenylalanine into new compounds. All of them have a general structure consisting of 3 phenyl rings and another heterocyclic ring containing an oxygen atom, forming a 15-carbon skeleton. Many of these compounds with variations in the two phenyl rings are found in nature [50]. Flavonoids are also present in the diets of people, reaching higher levels of daily intake than phenolic acids. Delving a little deeper into this group of compounds, flavonols are more common than flavones. They accumulate in the leaves and skin of plants and can act as complex-forming agents with metal ions due to the presence of carbonyl and hydroxyl groups in their structure [46].

## 4. Natural Antioxidant Mechanism in Radical Scavenging

Antioxidants have a differing capacity to stop the propagation of free radicals. The important factors influencing this are both the structure of the antioxidant and the structure of the compound to be oxidized, the presence of pro-oxidants, and the concentration of all of them. In addition, the region in the organism where all these substances are present and react together must be considered. There are various ways in which antioxidants carry out their work and there are numerous variables that can affect the antioxidant capacity [26]. This section will show some of the known mechanisms used by natural antioxidants in dealing with the propagation of free radicals. As the number of natural antioxidants is very large, only a selection of the best-known and most important ones will be discussed.

**Figure 2:**
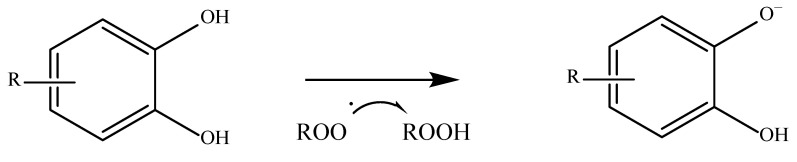
Phenolic acid derivative neutralizing a free radical via HAT [46].

Starting with phenolic acids, which are among the most abundant exogenous antioxidants, their ability to neutralize free radicals depends on several things, including the number of hydroxyl groups present on their aromatic ring, their position on it, and the presence of other substituents. As a general rule, the greater the substitution of the aromatic ring, the greater the difference in the antioxidant activity of these compounds. An aromatic ring that is unsubstituted cannot act as a hydrogen donor and therefore has a lower antioxidant capacity [51,52]. There are many different mechanisms by which an antioxidant compound can perform its function. The first and most common is known as HAT or hydrogen-electron transfer. This mechanism is used not only by phenolic acids but by all antioxidants that have a labile hydrogen atom in their structure. These compounds give up their hydrogen to stabilize the radical. Once this is carried out, the antioxidant compound becomes a radical itself, but it can be stabilized and reach a state where it is harmless [53]. A way of measuring how easily a hydrogen atom of an antioxidant compound can react with a free radical is the bond dissociation energy. The lower the value, the easier a hydrogen atom is transferred [54]. An example of this mechanism is shown in Figure 2.

**Figure 3:**
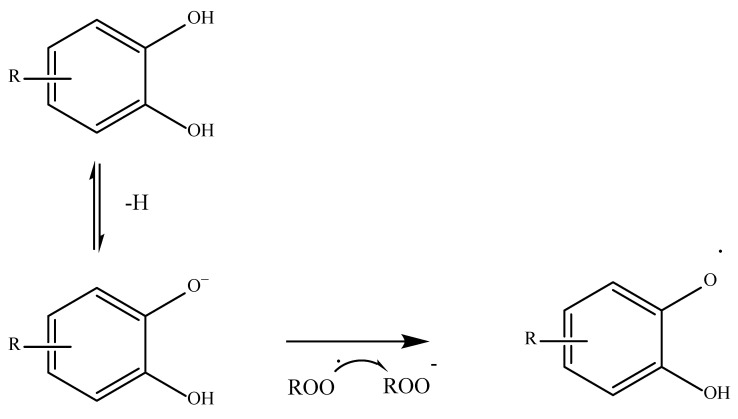
Phenolic acid derivative neutralizing a free radical via SET [46,53].

Another mechanism widely used by phenolic acids is the so-called SET or single electron transfer. In this case, the antioxidant gives up an electron to the free radical to stabilize it in an anionic form as shown in Figure 3 [46,53].

**Figure 4:**
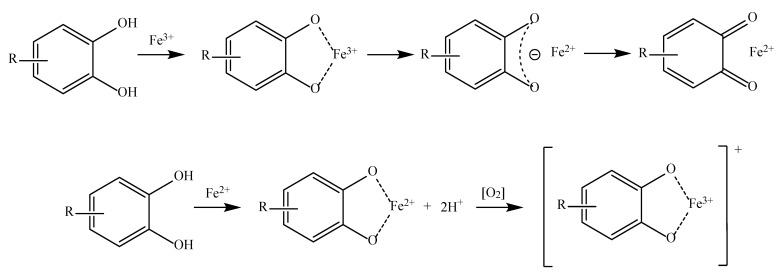
Phenolic acid derivative chelating Fe^3+^ and Fe^2+^ [46,53].

The third and fourth mechanisms by which the various phenolic acids exert their antioxidant capacity are known as transition metal chelation and sequential proton loss electron transfer (SPLET). The metal chelation mechanism is the ability of certain antioxidants to chelate transition metals, preventing them from catalyzing reactions that produce free radicals inside an organism (Figure 4). Some of these metals are Fe, Cu, and Mn [53,55].

**Figure 5:**
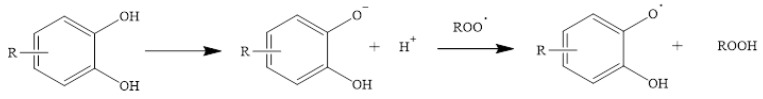
Phenolic acid derivative neutralizing a free radical via SPLET [53].

On the other hand, SPLET, or sequential proton loss electron transfer, occurs when the antioxidant compound donates a proton to a free radical and transforms into an anion, which subsequently donates an electron to stabilize itself [53]. An example of this is shown in Figure 5.

**Figure 6:**
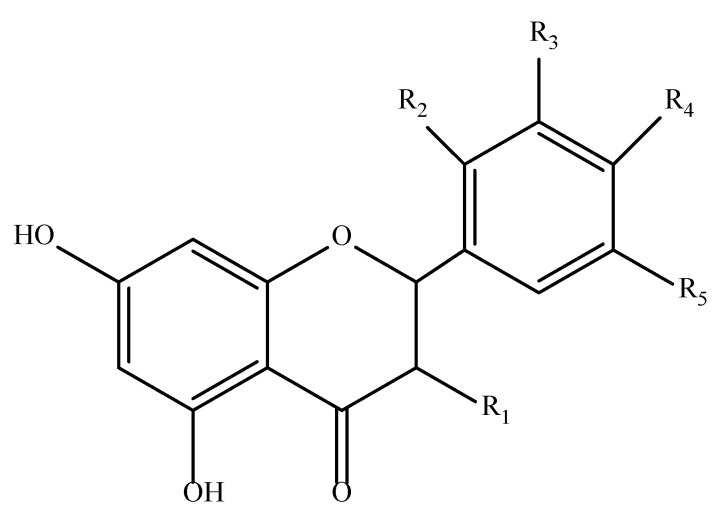
Most common structure of flavonoids. R1-R5 can range from hydrogen atoms to hydroxyl or methoxy groups [46].

In the previous section, it was pointed out that antioxidants derived directly from cinnamic acid have a higher antioxidant capacity than those derived from benzoic acid. This is due to the presence of the double bond of cinnamic acid, which can conjugate with the electron cloud of the aromatic ring, giving it a greater capacity to stabilize reactive species. Moreover, since the carbonyl group of cinnamic acid derivatives is distant from the aromatic ring, their antioxidant activity is higher than that of benzoic acid derivatives [26]. Flavonoids, as phenolic structures, also present these mechanisms. Their ability to neutralize free radicals is influenced both by the type of catechol ring present in their structure and by the presence of hydroxyl groups. Additionally, their double bond plays a critical role, as it can be conjugated and provide greater structural stability [46]. An example of a typical flavonoid structure is shown in Figure 6.

**Figure 7:**
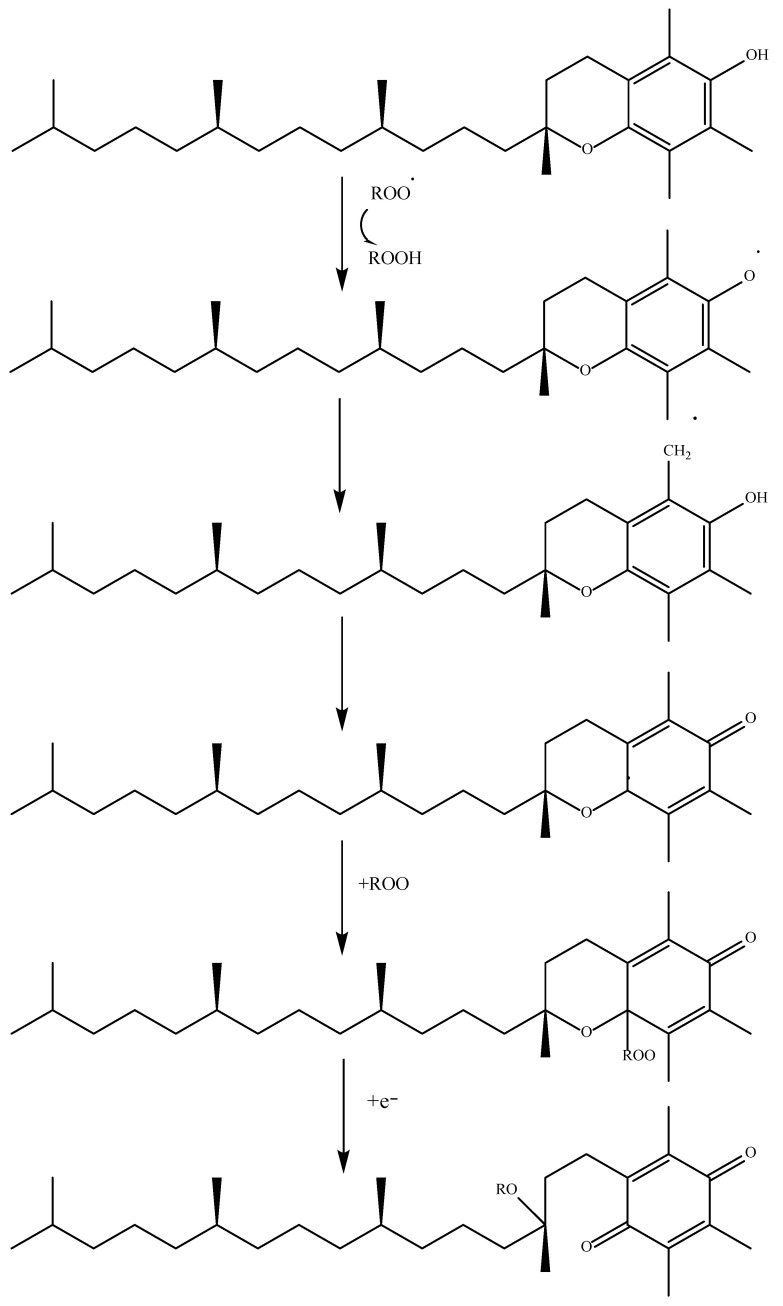
α-Tocopherol neutralizing a free radical via SPLET [46,49].

Apart from phenolic structures, there are many other compounds in exogenous antioxidants. Among vitamins, tocopherol, or vitamin E, is a fat-soluble vitamin present in cell membranes. Although this compound has a phenolic structure, it is classified within the group of vitamins. There are, in turn, different classes of tocopherols depending on their structure. α-Tocopherol is the most abundant form of vitamin E in nature [46,49]. This compound uses the SPLET mechanism to exert its antioxidant activity (Figure 7).

In the case of endogenous natural antioxidants, the non-enzymatic ones, such as transferrin and albumin, use the mechanism of metal chelation to trap metal ions as already mentioned. As for those that are enzymatic, such as SOD and CAT, they catalyze a series of reactions essential for the correct functioning of the organism [56]:

$$H_{2}O_{2}+\mathrm{Catalase}-\mathrm{Fe}\;\left(\mathrm{III}\right)\to H_{2}O+\mathrm{Catalase}-\mathrm{Fe}\;\left(\mathrm{IV}\right)\;H_{2}O+\mathrm{Catalase}-\mathrm{Fe}\;\left(\mathrm{III}\right)\to H_{2}O_{2}+\mathrm{Catalase}-\mathrm{Fe}\;\left(\mathrm{IV}\right)$$

## 5. Spectrophotometric Methods for Measuring Antioxidant Activity

Based on the previous discussion, it clear that it is critical to investigate the techniques that can be used to determine the total antioxidant activity (TAC) and total phenolic content (TPC). In this context, spectrophotometric (colorimetric and fluorescence) tests have received more attention as they are fast, reproducible, easy, and cheap [6,26,57]. Colorimetric assays, which are the most famous, such as DPPH, FRAP, ABTS, etc., change their color due to an electronic transition in atoms or molecules. A change in the electronic transitions affects how much light is absorbed by the molecules, which in turn alters the color of the molecules. Numerous complexes enter an excitation state after receiving an electron. The production of brightly colored complexes is caused by the fact that the excitation energy needed for an electron to transition from one energy level to another frequently falls in the visible area of the electromagnetic spectrum. Because of the different types of donors and acceptors involved, the absorption wavelength of the transition bands is unique. Any wavelength from 400 to 750 nm is visible as red, orange, yellow, green, blue, and violet [58]. All spectrophotometric methods are quantification techniques as a result of the regression line and regression equation of different concentrations of standards [59]. Depending on each case, the antioxidant structure and properties and the solubility and partition coefficient dictate the prevailing mechanism in a given system and guide the selection of the optimum assay [57,60]. Table 2 illustrates the spectrophotometric assays that will be addressed in more detail, along with their absorption maxima (λ_max_), fundamental principle, and any observed color shifting.

### 5.1. HAT and ET methods

#### 5.1.1. DPPH

In 1958, Blois [80] first devised this method by employing a stable free radical and using cysteine as a model antioxidant. This reaction is explained below, where Z• is used to simulate the DPPH radical and RSH the cysteine molecule. After the creation of RS, the free radical reacts with another molecule to produce RS-RS [81]:

$$Z^{•}+\mathrm{RSH}\to \mathrm{ZH}+{\mathrm{RS}}^{•}\;{\mathrm{RS}}^{•}+{\mathrm{RS}}^{•}\to \mathrm{RS}-\mathrm{SR}$$

Nowadays, DPPH is one of the most commonly used free radical scavenging antioxidant assays, which utilizes a π radical system of aromatic benzoic rings [26]. Due to the spare electron’s delocalization over the entire molecule, which prevents it from dimerizing like the majority of other free radicals, DPPH is classified as a stable free radical. It has been widely used in serum, biological fluids, and food samples. The only requirements are a UV spectrophotometer and DPPH reagent (2,2-diphenyl-1-picrylhydrazyl) [18]. DPPH reagent is a crystalline powder with a dark color, made up of stable free radical molecules, which is generally used as a radical and a trap (“scavenger”) for other radicals. Although the DPPH radical can be dissolved in several organic solvents, it cannot be dissolved in water. Methanol, ethanol, or aqueous mixtures of these compounds are typically used to dissolve it. The water quantity in this last case should not exceed 60% to make the radical more soluble [82].

Due to a broad absorption band centered at 515–520 nm (Table 2), the DPPH radical has a deep violet color in solution, and when neutralized, it becomes colorless or pale yellow [23]. The change in optical absorption can be used to measure the number of initiating radicals, and this characteristic allows visual monitoring of the reaction of ascorbic acid [59,83]. The EC_50_ value of the antioxidant activity of the DPPH scavenging method is defined as the effective antioxidant concentration required to reduce the initial DPPH concentration by 50%. It is also possible to use TEC_50_, which is the time it takes to reach the steady state with EC_50_. Another expression is the antiradical efficiency (AE) formula, which is described by Equation (1):

AE = (EC_50_ × TEC_50_)^−1^ (1)

This formula combines EC_50_ and TEC_50_ into a single quantity [27]. At the same time, Equation (2) is used to compute the reduction in the absorbance at various concentrations, which represents the antioxidant activity:

Antioxidant activity (%) = (E_radical_ − E_standard/_E_radical_) × 100 (2)

where E is the extinction coefficient of DPPH [81].

**Figure 8:**
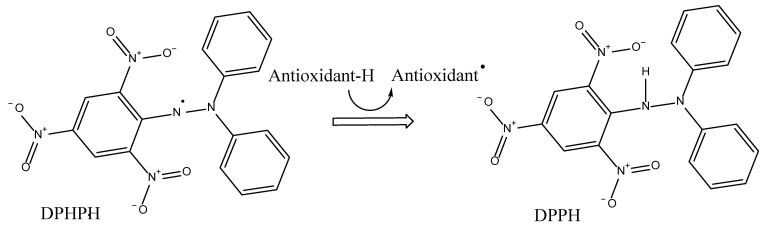
Theoretical mechanism of DPPH in the presence of an antioxidant [80,88].

DPPH has been used in the past in many studies to measure the antioxidant activity of common antioxidants such as ascorbic acid, BHT, propyl gallate, flavonoids, peptides, and phenolic acids [84,85,86,87], as it appears to have the potential to bind with any radical such as NO•, RS•, OH•, and O_2_•^−^ [81]. The currently proposed mechanism for DPPH is illustrated in Figure 8. It is believed that the mechanism is an electron transfer (ET) approach, as the hydrogen atom transfer (HAT) mechanism is merely a minor reaction pathway [60].

Furthermore, DPPH is characterized by many advantages as it is simple, inexpensive, and fast in contrast with other assays such as ABTS. The radical is stable and does not require a generator. Even with weak antioxidants, the radical scavenging period of 30 min enables DPPH to react effectively. To prevent the possibility of thermal destruction of the compounds being examined, the antioxidant efficiency is assessed at room temperature. The results are repeatable and extremely accurate. Additionally, DPPH can quickly screen a large number of samples and bioactive substances (polyphenols, flavonoids) with a good correlation. However, other radicals in the examined substances tend to react with DPPH. In addition to this, Lewis bases have an impact on DPPH and the absorbance tends to decrease when exposed to light [58,81]. Finally, various modifications of the DPPH method have been involved for a wide range of applications based on the requirements and, more importantly, affordable inputs.

#### 5.1.2. Folin–Ciocalteu (FC)

**Figure 9:**
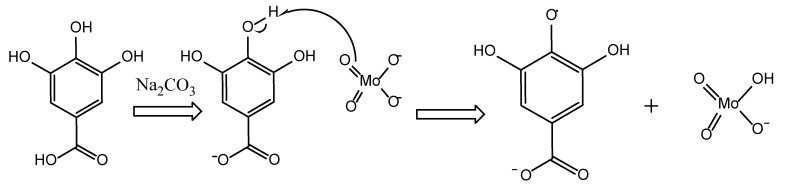
Folin reagent transformation mechanism with gallic acid [96].

Folin–Cioclteu (FC) reagent is a mixture of phosphomolybdate and phosphotungstate used for the colorimetric in vitro analysis of phenolic and polyphenolic antioxidants [89]. The name Folin–Cioclteu was given after Otto Folin, who first proposed the quantification of tyrosine levels in 1927 [62,90]. It is the most commonly used assay for identifying the total phenolic content of diverse plant or food samples. FC generates a blue color when it reacts with phenols and is absorbed at 760–765 nm. The blue color is thought to be caused by a complicated Mo(V) [62]. The assay that was initially created is the Folin–Denis (F-D) test, which was then used to assess the total protein concentration by measuring the tryptophan and tyrosine levels. However, during the process of improvement, the FC assay was found to be more accurate and repeatable. Thus, it has been used over the years not only for the determination of total phenolic content but also for the determination of nitrogen-containing substances such as hydroxylamine and guanidine, thiols, numerous vitamins, the nucleotide base guanine, the trioses glyceraldehyde and dihydroxyacetone, flavonoids, and various inorganic ions [60,62,91,92,93]. The current theoretical oxidation–reduction mechanism is SET or ET. The original FC assay uses a buffer for pH adjustment while gallic acid is the most commonly used reference standard. TPC values are usually given as gallic acid equivalents (GAE) [59,63,89]. However, TPC values are sometimes presented as catechin, caffeic acid, chlorogenic acid, or ferulic acid equivalents [94]. As Figure 9 demonstrates, the reaction between FC reagent and phenolic compounds occurs at pH 10 due to the addition of Na_2_CO_3_. In these alkaline conditions, the dissociation of phenolic proton results in the creation of a phenolate ion, which is in charge of reducing the FC reagent. Hence, the central molybdenum ion accepts one electron from the phenolic antioxidant, and hence, the reduction from Mo^6+^ ion to Mo^5+^. The anionic derivatives of the phosphotungstic and phosphomolybdic acids change from a yellow color to blue [95].

The use of the Folin–Ciocalteu test to quantify TPC has numerous benefits, including ease of use, repeatability, and robustness. It does, however, have significant shortcomings. The test is sensitive to pH, temperature, and the reaction duration. Therefore, carefully selection of the reaction state is crucial for obtaining consistent and trustworthy findings. Due to the contribution of the non-phenolic reducing agents present in the system when reducing Folin–Ciocalteu reagent, TPC overestimation is a significant concern for the Folin–Ciocalteu test [58]. Reducing sugars and certain amino acids are two examples of these compound categories. As a result, when compared to data using HPLC techniques, TPC measurement results may be overstated [26].

#### 5.1.3. CUPRAC

Recently, Apak and his group developed, for the first time, the cupric ion-reducing antioxidant power (CUPRAC assay, 2,9-dimethyl-1,10-phenanthroline) [97]. CUPRAC tests an antioxidant’s ability to decrease an oxidant, which changes color when it is reduced. The degree of color change is proportional to the total antioxidant capacity concentration as cupric Cu^2+^ transforms to cuprous Cu^+^ [26]. This approach has been utilized at 450 nm and it has been used with both lipophilic and hydrophilic antioxidants such as epicatechin gallate, rosmarinic acid, quercetin, epigallocatechin, catechin, acid caffeic acid, epicatechin, gallic, rutin, and chlorogenic acid in diverse matrices [5,98]. Further antioxidants measured by the CUPRAC method are ascorbic acid, tocopherol, carotene, uric acid, albumin, and bilirubin [5].

**Figure 10:**
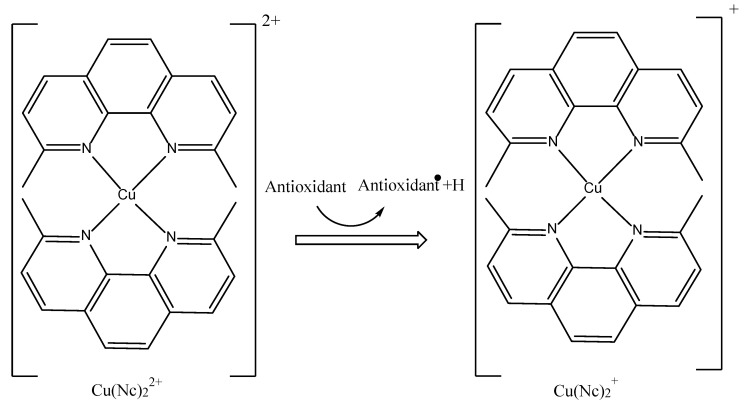
Proposed CUPRAC general reaction mechanism [23,26,46].

There are many different phenanthroline derivatives, such as neocuproine (Nc), BCS (2,9-dimethyl-4,7-diphenyl-1,10-phenanthroline disulfonic acid), and bicinchoninic acid (BCA:2-(4- carboxyquinolin-2-il) chinolin-4-carboxylic acid), which can stabilize Cu^+^ ions [46,64,65,99]. The transition of Cu(Nc)_2_^2+^ to Cu(Nc)_2_^2+^ changes the color from light blue to yellow [26]. A general explanation of the current reaction is illustrated in Figure 10. Usually, CUPRAC is utilized at a pH of 7.0 with ammonium acetate aqueous buffer, which is close to the physiological pH of 7.4 and takes around 30 to 60 min depending on the speed of the antioxidant [26]. The proposed mechanism is explained by electron transfer (ET) or single electron transfer (SET), along with the FRAP, DPPH, and Folin approaches [81,100]. The CUPRAC capacity can be expressed either as % inhibition or the equivalent of a standard compound, namely Trolox, gallic acid, ascorbic acid, quercetin, or α-tocopherol. Some of CUPRAC´s biggest advantages are that the reagents are more widely available, less expensive, and more stable than DPPH. It can work with simple instrumentation, and there have been no reports of chemical interference in solutions. In fact, there is always a good correlation with many polyphenolics, including flavonoids and phenolic acids [5,81,101].

#### 5.1.4. FRAP

Ferric-reducing/antioxidant power (FRAP) is another colorimetric method that was first announced by Iris Benzie and J.J. Strain for the quantification of ascorbic acid in plasma and serum [102]. Since then, it has been widely used to measure chalconaringenin, rutin, ascorbic acid, chlorogenic acid, lycopene, and phenolic compounds in a variety of matrices such as vegetables, herbs, fruits, beverages, oils, and medicinal plants [23]. The transformation from a colorless solution to a blue solvent is caused under acidic conditions with pH of 3.6 to maintain iron solubility while the maximum intensity is observed at 593 nm [23,26,68]. Lower pH levels reduce the ionization potential, which drives electron transfer while increasing the redox potential, resulting in a shift in the primary reaction mechanism [26]. Even though the FRAP assay provides quick, repeatable findings, there is one major limitation as the antioxidant must be water soluble [102].

**Figure 11:**
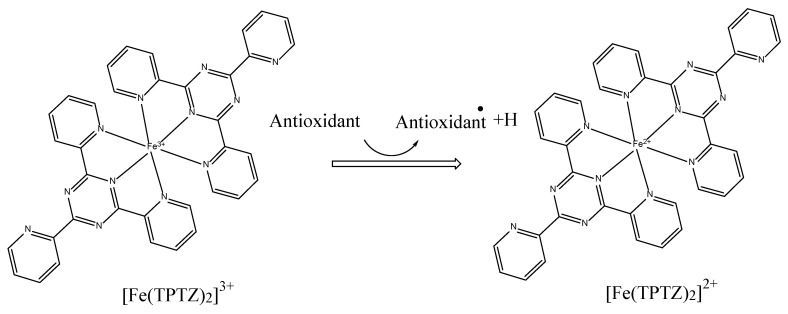
FRAP reductive mechanism by antioxidant species [26].

When Fe^3+^-TPTZ (ferric 2,4,6-tripyridyl-s-triazine) complex reacts with an antioxidant compound, a single electron is transferred to the ferric ion, converting it into Fe^2+^-TPTZ (ferrous tripyridyltriazine). Therefore, the FRAP method can again be described as an ET-based or SET-based mechanism [26]. A possible representation of the general mechanism with [Fe(TPTZ)_2_]^3+^ is given in Figure 11.

Although tripyridyltriazine (TPTZ) is the iron-binding ligand in the original FRAP assay, other ligands have been used for ferric binding, such as ferrozine for ascorbic acid–reducing power evaluation. More recently, FRAP investigations have suggested potassium ferricyanide laggard (PFRAP) as a new promising ferric complex [26,57]. As Prior et al. reported that other antioxidant activity assays have a weak association with FRAP, it is advised that this assay is used in conjunction with other approaches to identify dominant pathways for various antioxidants [60]. Nevertheless, FRAP is characterized by low cost, sensitivity, and repeatability, as it is capable of screening a broad range of biological materials, including aqueous and organic extracts from pharmaceuticals, foods, and plants, in addition to plasma, blood, serum, saliva, tears, urine, cerebrospinal fluid, and exudates and transudates [58,60].

#### 5.1.5. FOX

The ferrous oxidation xylenol orange (FOX) reagent is an aqueous solution of ferrous ammonium sulfate, sorbitol, sulfuric acid, and xylenol orange that is used to assess hydrogen peroxide levels mostly in biological systems and sometimes in plants. After a series of oxidation processes, the reagent is incubated with the sample, and the absorbance is measured at a wavelength of 560 nm [72]. FOX is utilized in an acidic environment and the test relies on the oxidation of Fe^2+^ to Fe^3+^ [73]. An oxidant, such as hydroperoxides (see the below reaction), oxidizes ferrous ion to ferric ion, which is then treated with xylenol orange (XO) reagent to generate a ferric-XO complex, which gives a blue–purple color at 550–560 nm [23]. The current procedure has received significant attention due to its low cost but also because FOX reagent is unaffected by environmental conditions such as oxygen or light levels. It is still widely used to determine hydroperoxides in various biological samples and lipoxygenase activity in plant extracts and plant tissue [23]. However, this assay has only been applied in a few investigations into natural antioxidants:

$${\mathrm{Fe}}^{2+}\overset{\mathrm{Hyperoxides}}{\to }{\mathrm{Fe}}^{3+}\overset{\mathrm{Xylenol}\;\mathrm{Orange}}{\to }{\mathrm{Fe}}^{3+}\;“\mathrm{Xylenol}\;\mathrm{Orange}\;\mathrm{Complex}”\;$$

#### 5.1.6. FTC

The mechanisms associated with the ferric thiocyanate (FTC) assay are identical to FOX, with the only difference being that ferric ion is converted by an oxidant from ferrous ions that are monitored as a thiocyanate complex distinguished at 500 nm [23]. The combination of Fe^3+^ and [SCN]^−^ ions has a blood-red color. The ferric thiocyanate assay determines the presence of different oxidizers, such as lipid hydroperoxides, and evaluates the effects of antioxidants by oxidizing ferrous to ferric ions and then complexing the latter with thiocyanate. The current process is referred to below. Most investigations, including the FOX assay, are performed at a stable pH value of 7.0 and 40 °C while the absorbance is measured at 500 nm every 24 h until it reaches a maximum value. This is a simple and repeatable experiment that is commonly used for the identification and quantification of the total phenolic content, lipid oxidation, and flavonoid content. Usually, gallic acid is used as a standard, but if any chemical is absorbed in the area of 500 nm, the results are overestimated or unreliable [23,74,103]:

$$\mathrm{Fe}{\left(\mathrm{SCN}\right)}_{2\;}\;“\mathrm{FTC}\;\mathrm{Reagent}”\overset{\mathrm{Hyperoxides}}{\to }\mathrm{Fe}{\left(\mathrm{SCN}\right)}_{3}\;“{\mathrm{Fe}}^{3+}\;\mathrm{Thiocyanate}\;\mathrm{Complex}”$$

#### 5.1.7. β-Carotene Bleaching Assay

**Figure 12:**
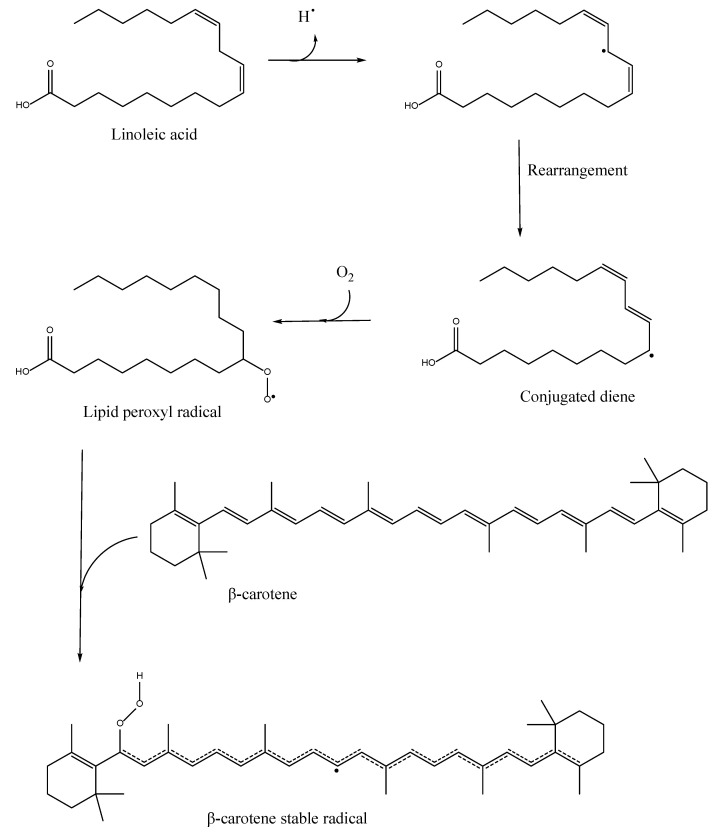
β-carotene stabilization mechanism [58].

The initial application for this assay was proposed by Marco et al. [104] in 1968 in an attempt to prevent the autooxidation of emulsified linoleic acid in extracts. Over time, adjustments were made to the technique to increase the convenience and reduce its flaws. The current assay can quantify pro-oxidants and antioxidant species. However, this application is extensively applied to peroxyl radicals to produce β-carotene epoxides, which act as radical scavengers or antioxidants [75]. Lipids, such as linoleic acid, form a peroxyl radical (LOO•) in the presence of ROS and O_2_. As shown in Figure 12, this peroxyl radical combines with β-carotene to create a stable carotene radical. As a result, the concentration of β-carotene in a testing solution is reduced. In the presence of an antioxidant, β-carotene competes for the formation of the mentioned adduct. Antioxidant effects can thus be easily verified by bleaching the color of a test solution at 470–490 nm [23,97].

The noticeable difference in these nanometers is the decolorization of the orange–yellow or dark-yellow carotene solution, which is caused by breaking the π-conjugation by the addition reaction of radicals into a C=C bond of β-carotene. The bleaching rate (R) of β-carotene is measured with Equation (3):

R = [ln(a/b)/t](3)

where ln = natural log, a = absorbance at time 0, and b = absorbance at time t. Additionally, the antioxidant activity can be measured with Equation (4) [58]:

AA = (R_control_ − R_sample_)/R_control_ × 100 (4)

Many different phenolic compounds have been confirmed with this assay, along with flavonoids and lipid acids. However, because of the differences in the antioxidant molecule sizes, β-carotene activity sometimes lacks linearity. The lipophilicity of phenolic antioxidants increases as the chain length increases, leading to an increase in the antioxidant activity up to a certain point. A sharp decrease in the antioxidant activity is observed after a particular chain length. This threshold phenomenon is referred to as the cut-off effect [105]. Therefore, based on this theory, high lipophilicity derivatives have a higher inhibitory effect on β-carotene bleaching. As a result, it is crucial to test how well the antioxidants work in oil–water emulsions to assess their characteristics before being used in food systems [58].

The current assay can screen materials that are both lipophilic and hydrophilic. However, many drawbacks must be considered, as it is time-consuming and it has a poor correlation with several assays, including FRAP, CUPRAC, ABTS, and DPPH. Moreover, β-carotene is highly susceptible to the effects of oxygen, pH, temperature, and solvents [58] while the reproducibility of this assay varies significantly according to Prieto et al. [106] and Mikami et al. [107], who reported high and low reproducibility, respectively.

#### 5.1.8. ABTS

The Trolox equivalent antioxidant capacity (TEAC) or ABTS, as it is also well known as, was first developed by Miller et al. [108] for measuring the total antioxidant capacity in body fluids and drug solutions based on the absorbance of the ABTS•^+^ radical cation. More specifically, this assay assesses antioxidants’ ability to scavenge the stable radical cation ABTS•^+^ (2,2′-azinobis (3-ethylbenzothiazoline-6-sulfonic acid). An ABTS•^+^ radical is generated when ABTS is converted by a strong oxidant. Metmyoglobin and hydrogen peroxide were employed in the original TEAC test to produce an intermediate radical of ferrylmyoglobin, which then interacts with ABTS to produce ABTS•^+^. Years later, peroxide or persulfate was used to replace the oxidizing agent [26].

**Figure 13:**
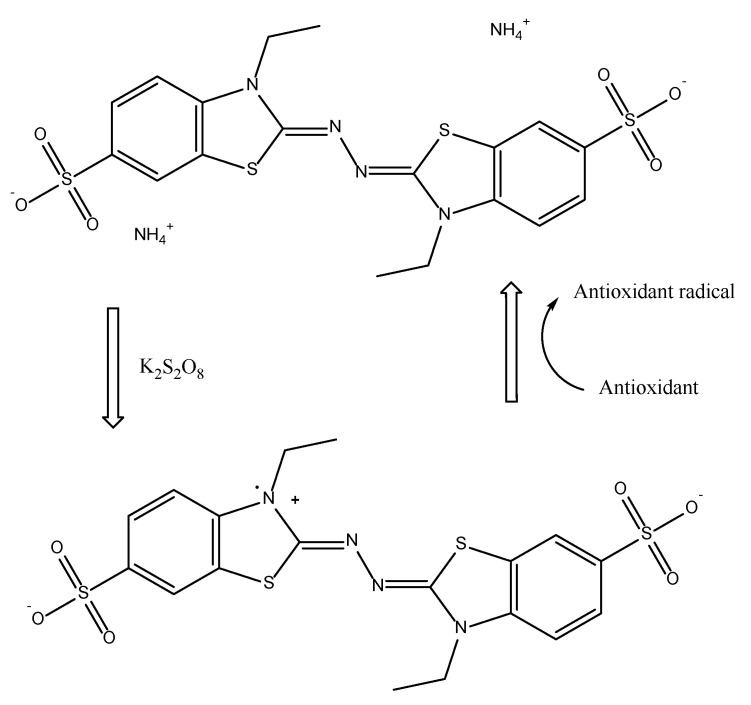
Proposed ABTS mechanism [26,101].

Antioxidants can neutralize the radical cation produced by ABTS by either direct reduction by electron donation or radical quenching via hydrogen atom donation, with the balance of these two pathways [27]. As a result, while the TEAC assay is typically classified as an ET-based approach, the HAT mechanism is equally applicable. Figure 13 describes the formation of ABTS•^+^ from ABTS using K_2_S_2_O_8_ followed by the reaction with antioxidant species. During this process, a blue–green chromophore with maximum absorption at 734 nm is produced in the presence of antioxidants. Among these, 734, 414, 645–650, 734, and 815–820 nm are also reported by Opitz et al. [67], Prior et al. [60], and Rubio et al. [64], as the maximum absorption depends on the reaction duration, intrinsic antioxidant activity, and sample concentration. As the blue–green ABTS•^+^ chromophore can be absorbed at several different wavelengths, 734 nm is selected as the optimum in many publications since any potential interferences are minimized and decreased. The current assay evaluates antioxidant mechanisms in dietary components in a wide pH range on both lipophilic and hydrophilic compounds in water and organic solvents [26].

Using Formula (5), the Trolox equivalent antioxidant capacity can be measured:

TEAC_sample_ = (A_sample_ − A_blank_)/(A_Trolox_ − A_blank_). f. C_Trolox_, w (5)

where A_Trolox_ represents the maximum absorbency after the addition of an established volume of Trolox, A_sample_ represents the maximum absorbency measured after the addition of an established volume of sample, A_blank_ represents the maximum absorbency measured at end-point addition after the addition of an established volume of solvent, f is the dilution factor of the sample, and C_Trolox_ is the effective concentration of Trolox in μmol/l [109].

Nowadays, the ABTS or TEAC test is more commonly used for measuring the relative ability of antioxidants to scavenge ABTS generated in the aqueous phase, as compared with a trolox (6-hydroxy-2,5,7,8-tetramethylchroman-2-carboxylic acid) standard. A wide range of free radicals, including hydroxyl, peroxyl, alkoxyl, and inorganic radicals, react quickly with ABTS [57,60]. The diphenylpicrylhydrazyl (DPPH), oxygen radical absorbance capacity (ORAC), and ferric-reducing ability of plasma (FRAP) assays are some other antioxidant capacity assays that employ Trolox as a standard. Frequently, TEAC is used to determine the antioxidant content of foods, drinks, and dietary supplements [24]. Previous articles have also used this assay on aromatic plants, carotenoids, and phenolic compounds.

In contrast to the DPPH radical, which dissolves solely in an organic medium, the ABTS cationic radical is soluble in both organic and aqueous media. Because of this, both lipophilic and hydrophilic materials can be screened using the ABTS assay. Additionally, a good correlation has been reported for bioactive compounds (phenols, flavonoids), with a regression factor of more than 0.8. On the other hand, one major disadvantage is that the ABTS^+^ radical cannot be produced chemically and cannot be found in any biological system. ABTS^•+^ radical production is a slow reaction that takes between 12 and 16 h as opposed to the easily accessible commercial DPPH [26,58].

#### 5.1.9. ORAC

The oxygen radical absorbance capacity (ORAC) was founded by Ghiselli and Glazer et al. [60] and was developed further by Cao et al. [110]. Since then, the ORAC test has been established in food, pharmaceutical, botanical, biological, and cosmetic samples because it can be used to indicate any oxygen species. The fundamental principle of ORAC depends on the fluorescence quenching that occurs when antioxidants react with a peroxyl radical. There are many peroxyl radical generators such as α-azobisizobutyronytril (AIBN), 2,2-azobis(2-amidinopropane) chlorhydrate (ABAP), and 2,2-azobis(2,4-dimethylvaleronytril) (AMVN); however, 2,2′-azobis(2-amidinopropane) dihydrochloride (AAPH) is most commonly used [111].

**Figure 14:**
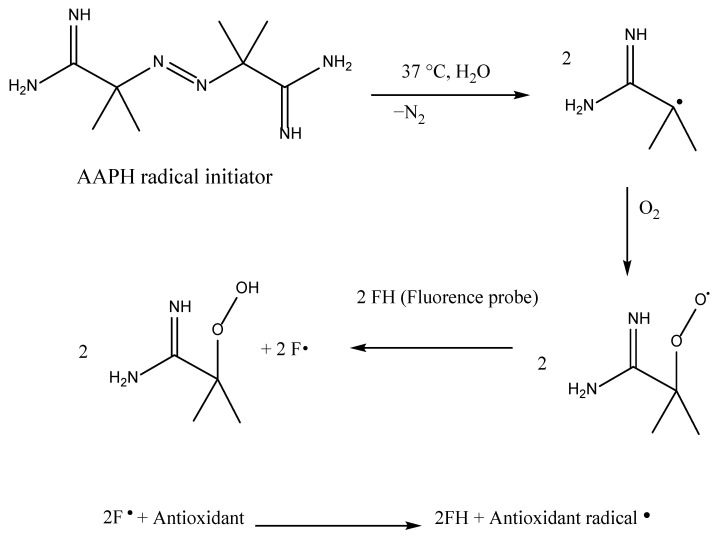
AAPH generates free radicals and afterwards, the reaction between the fluorescence probe and antioxidant occurs [23,112].

The main mechanism is described in Figure 14, showing the interaction of the thermally generated C-centered free radicals produced by AAPH with the fluorescent probe as peroxy-free radicals when oxygen is present. The oxidizable substrate/fluorescent probe can react with the peroxyl radical, modifying the fluorescence intensity and typically speeding up the rate of fluorescence degradation. The azo AAPH utilizes peroxyl radical generators in hydrophilic systems, with phycoerythrin or, more recently, fluorescein as the fluorescent probe. In the case of the evaluation of hydroxyl radicals, H_2_O_2_–CuSO_4_ is commonly employed while phycoerythrin is a redox-sensitive fluorescent indicator protein whose fluorescence degradation is evaluated in the presence of free radical scavengers using Trolox as a standard [26,60]. Usually, the measurements are expressed as Trolox equivalents per gram on a dry basis [26,60,68]:

AC_Fl_ = [(AUC_Sample_ − AUC_Blank_)/(AUC_Trolox_ − AUC_Blank_)] × (molarity of Trolox/molarity of sample)

where AC_Fl_ represents the antioxidant capacity, AUC_sample_ represents the net area under the curve of the mixture, AUC_blank_ is the net area under the blank curve buffer, and AUC_Trolox_ is the net area under the curve of Trolox [60,68].

The ORAC assay is related to the study of ascorbic acid, bilirubin, glutathione (GSH), uric acid, α-tocopherol, β-carotene, and polyphenols [97]. The first trial employed B-phycoerythrin (B-PE), a protein obtained from *Porphyridium cruentum* [68]. Major drawbacks include the inconsistency in the assay results and false low ORAC values of B-PE photobleached after exposure to excitation light. Therefore, these reasons led to the development of more stable complexes that are less reactive FL(3′,6′-dihydroxy-spiro[isobenzofuran-1[3H],9′[9H]-xanthen]-3-one) or dichlorofluorescein (H_2_DCF-dA-2′,7′-dichlorodihydrofluorescein diacetate) [24,68,110]. The ORAC_FL_ assay was designed to measure the hydrophilic chain-breaking antioxidant capacity against peroxyl radicals. This excludes lipophilic antioxidants, which are particularly crucial against lipid oxidation in all systems, and other physiologically reactive radicals (HO•, HOO•, ONOO•, O_2_•^−^, etc.). To make the ORAC assay more widely applicable, it was modified to assess both lipophilic and hydrophilic antioxidants using a solution of acetone/water (50 % *v*/*v*) containing 7% randomly methylated-cyclodextrin (RMCD) to solubilize the antioxidants [10].

ORAC and ORAC_FL_ are described as a traditional HAT reaction mechanism [67]. Usually, the oxidation of fluorescein is accompanied by a decrease in the fluorescence measured over time at an excitation wavelength of 485 nm and an emission wavelength of 520 nm for 30–40 min at pH 7.4 and 37 °C [26,68]. This change in fluorescence is dependent on the number of free radicals. It is also worth mentioning that when the pH value drops below 7, its intensity decreases significantly.

The HORAC assay was developed by Ou et al. (2002) [113] as there was a validated assay for the total antioxidant capacity for peroxyl radicals (ORAC) but no such assay had been reported for hydroxyl radicals in matrices such as biological fluids, cells, plants, and tissue. HORAC uses a Co (II)-mediated-Fenton mixture (Co (II), Fe (II), and H_2_O_2_) to generate a hydroxyl radical. The formation and hydroxylation of p-hydroxybenzoic acid confirms the generation of the hydroxyl radical under the experimental conditions. The original fluorescence readings are obtained every minute after stirring. Different quantities of gallic acid standard solutions are used to create the calibration curve. The hydroxyl radical can oxidize fluorescein (3,6-dihydroxy-spiro[isobenzofuran-1[3H],9[9H]-xanthen]-3-one) to produce a fluorescein-free product.

Antioxidants suppress this reaction by a hydrogen atom transfer mechanism, inhibiting the oxidative degradation of the fluorescein signal. The fluorescence signal is measured at an excitation of 485 nm and emission occurs at 535 nm in the same conditions as ORAC (pH 7.4 and 37 °C). The diluted sample is reanalyzed until the fluorescent reading is reduced by more than > 95%. The HORAC values are expressed as micromoles of GAE per gram for solid samples and as micromoles of GAE per liter for liquid samples [113]. The area under the fluorescence decay curve (AUC) is integrated, and the net AUC, a measure of the hydroxyl radical prevention capacity, is obtained by subtracting AUC of the blank from that of the antioxidant [24,114]:

Fl = [(AUC_sample_ − AUC_blank_)/(AUC_gallic acid_ − AUC_blank_)] × (molarity of gallic acid/molarity of sample)

Although the actual mechanism of the Fenton-like reaction is exceedingly complex and there is no solid evidence of how hydroxyl radicals are involved, it is believed that HORAC uses an HAT mechanism [26,115].

#### 5.1.10. TBA-TBARS

From the earliest 1940s to 1996, thiobarbituric acid (TBA) was a very common spectrophotometric procedure for monitoring the transformation of aromatic aldehydes, 2-deoxy sugars, and HO• radicals at 535 nm [69]. Nowadays, the TBA assay is especially well-suited for the detection of oxidative rancidity in lipids, which are unsaturated and contain two or three bonds. In particular, it is useful for matrices such as fruits and vegetables or biological fluids [71]. There are no previous reports related to medicinal plants [70,101]. This technique involves the combination of ascorbic acid (AA), deoxyribose, phosphate buffer, ferric chloride, hydrogen peroxide, ethylenediamine tetraacetic acid (EDTA), trichloroacetic acid (TCA), and thiobarbituric acid (TBA). Each of these reagents plays a key role in the assay.

**Figure 15:**
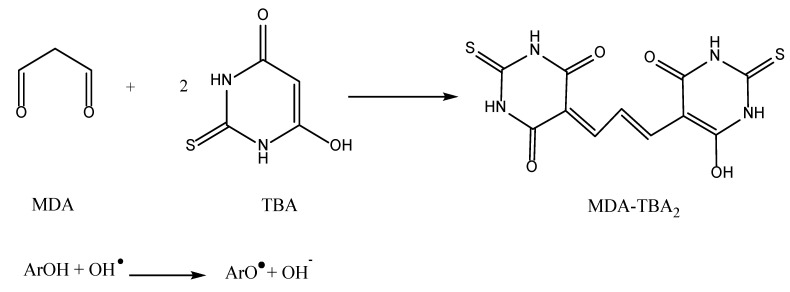
Presentation of the formation reaction of MA-TBARS [26,101].

The test begins by complexing EDTA with Fe^2+^, which interacts with H_2_O_2_ to produce the HO• radical. The radical must be produced at a temperature of 37 °C for approximately 12 h. In the presence of ascorbic acid, the produced HO• radical reacts with the deoxyribose sugar to produce a variety of products. The addition of ascorbic acid is intended to speed up the radical’s breakdown of deoxyribose in the reaction mixture. When the resulting product mixture with TBA is heated at a low pH value, it forms malondialdehyde (MDA). After the creation of MDA, the MDA-TBA chromogen is formed. As demonstrated in Figure 15, a nucleophilic attack involving TBA and MDA is followed by dehydration to produce the final solvent of MDA-TBA_2_, also known as TBARS. The scavenging activity toward the HO• radical is measured based on the inhibition of deoxyribose degradation.

Generally, the MDA/TBA ratio is a good predictor of lipid peroxidation. The oxalate toxicity produced by the enhanced lipid peroxidation is indicated by elevated levels of thiobarbituric acid reactive substances (TBARS). The color of TBARS solvent is red-pink and can be detected spectrophotometrically at 532 nm. Normally, the TBARS assay takes around 2 h to be completed and is carried out at 50–70 °C in an acidic environment (pH = 4) [70]. However, because MDA is extremely water soluble and tends to appear as a polymer in an aqueous solution, its detection in a lipid sample is particularly challenging. The TBA number is defined as the number of milligrams of malonaldehyde per kilogram of sample [101].

### 5.2. Targeted Scavenging Activities

#### 5.2.1. Hydrogen Peroxide Scavenging Assay Activity

**Figure 16:**
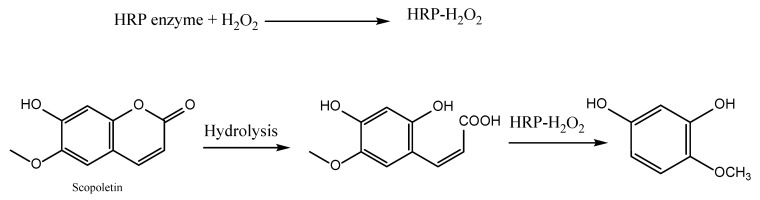
General mechanism of hydrogen peroxide scavenging [58].

Horseradish peroxidase (HRP) is the most frequently employed enzyme in this test. Typically, an HRP-H_2_O_2_ complex reacts with 7-hydroxy-6-methoxycoumarin (scopoletin), which is a fluorescent substrate. This process is described in Figure 16. Under UV light, the blue color of the product can be detected at 460 nm at pH 4.5. Borate buffer is used to inhibit the reaction (pH 10) [116]. The reaction is stopped, and the fluorescence or absorbance is then determined. Either spectrophotometric or fluorometric approaches can be used to measure the scavenging capacity. The fluorescence intensity is inversely proportional to the concentration of oxidized scopoletin. Therefore, the amount of H_2_O_2_ can be determined by observing the decrease in scopoletin’s fluorescence [58,117].

#### 5.2.2. Superoxide Radical Scavenging Activity

**Figure 17:**
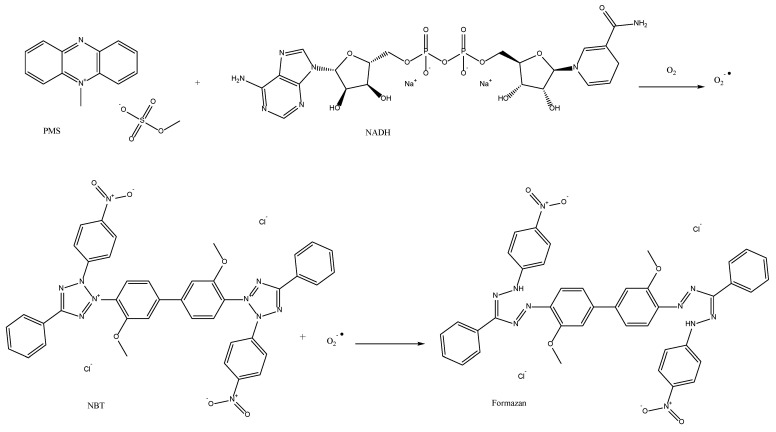
NBT mechanism reacting with superoxide radicals [58].

Superoxide dismutase or SOD, as it is well known, is the name of the antioxidant enzyme that is responsible for scavenging O_2_•^−^ radicals. This enzyme was discovered in 1969 by McCord and Fridovich [118]. SOD has the ability to transform O_2_•^−^ radicals into H_2_O_2_, which is then broken down by catalase and glutathione peroxidase into O_2_ and water. One of two methods that can be used to produce O_2_•^−^ radicals is a non-enzymatic method employing phenazine methosulphate (PMS), nitroblue tetrazolium (NBT), and a reduced version of nicotinamide-adenine dinucleotide (NADH). The other is described by Robak and Gryglewski [119] and is based on a hypoxanthine-xanthine oxidase superoxide-generating system. Figure 17 illustrates the first assay mechanism, with the invention of NBT, as it is the most famous one. NBT is a light-yellow soluble salt before it is reduced by oxygen. When the reduction occurs at pH 7.4, the tetrazole ring is broken, causing dismutation, which then produces a bright blue color product called formazan [58]. Superoxide radicals are produced by the non-enzymatic phenazine methosulfate-nicotinamide adenine dinucleotide (PMS/NADH) system. The reaction has maximum absorbance at 560–562. This application has been widely used for many antioxidants and especially biological samples [76,77]. However, it cannot be characterized as a specific and targeted assay since different reductases can lower NBT.

#### 5.2.3. Nitric Oxide Radical Scavenging

**Figure 18:**
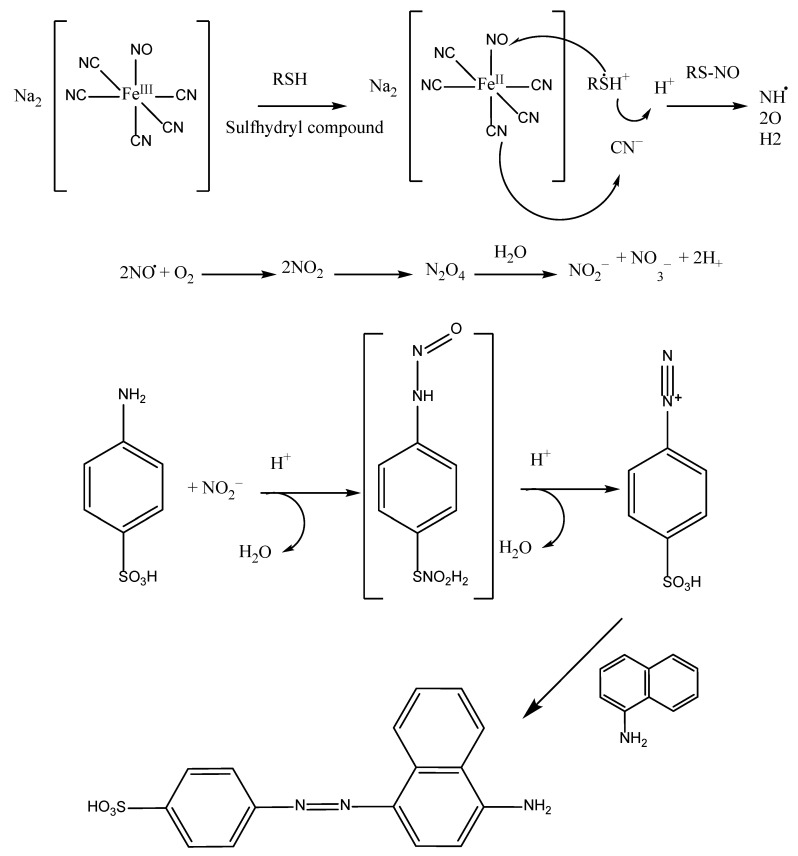
Mechanism reaction of how NO free radicals are scavenged by Griess reagent [58].

Nitric oxide radicals have been determined by aqueous sodium nitroprusside (SNP) solution, which can react with oxygen at physiological pH 7.2 to form nitrite ions. These nitric ions can therefore be measured using the Griess–Illosvoy method [120]. Johann Peter Griess, a German scientist, invented the diazotization assay in 1864. The modified experiment featured the interaction of nitrite (NO_2_^−^) with sulfanilic acid in an acidic environment. This produced a diazonium ion, which was then linked with N-(1-naphthyl) ethylenediamine (NED) to produce a water-soluble and red azo dye (HO_3_SC_6_H_4_-NN-C_10_H_6_NH_2_). The addition of NED is necessary to improve coupling and the repeatability, sensitivity, and solubility of the azo molecule. The red color that occurs is indicated at 540 nm against a blank sample [76]. Furthermore, under aerobic conditions, NO• can combine with O_2_ to form the stable compounds nitrate (NO_3_^−^) and nitrite (NO_2_^−^), which can also be measured using the Griess reagent. However, the synthesis of NO_3_^−^ and NO_2_^−^ does not if an antioxidant is present [58] (Figure 18).

#### 5.2.4. Peroxynitrite Scavenging Capacity

**Figure 19:**
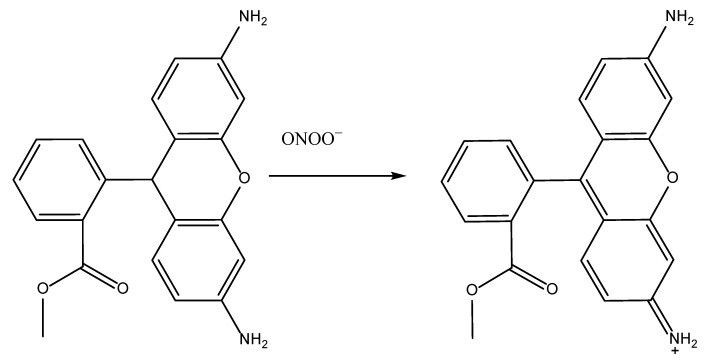
Peroxynitrite reaction with dihydrorhodamine (DHR) [58].

Only a few articles have been published about the ability of an antioxidant to scavenge peroxynitrite radicals. The two most important methods are, namely, the inhibition of tyrosine nitration by ONOO^−^ [121] and the inhibition of dihydrorhodamine (DHR) at wavelengths of 485 or 505 and 529 or 530 nm, respectively, in the presence of an antioxidant [23,58]. Figure 19 describes the general mechanism involving the DHR reagent and free radical. Nevertheless, the DHR method has one major limitation: it lacks specificity. As ONOO^−^ quickly breaks down, NO• and O_2_•^−^ are produced and DHR could potentially be oxidized by these two species. For this reason, Beckman et al. [122] first described a method for synthesizing peroxynitrite and measuring its concentration spectrophotometrically at 302 nm. With further developments by Evans Blue [78], the peroxynitrite scavenging activity is measured at pH 7.4 at 611 nm after 30 min of incubation at 25 °C. By comparing the findings of the test and blank samples, the percentage scavenging of ONOO^−^ can be determined.

## 6. Advantages and Limitations of Spectrophotometric Assays

Antioxidant scavenging is becoming more interesting to scientists, who are attempting to understand the mechanisms involved in biological systems, the antioxidant capacity of food, and the free radicals of various substrates. Alternative methods for the measurement of antioxidant activity are still required (liquid and gas chromatography, electrophoretic procedures, etc.), although spectrophotometric applications are important. The benefits of employing spectrophotometric TAC assays are numerous and include their ease of use, low cost per sample, quick turnaround times, and the ability to be carried out manually, semi-automatically, or automatically. However, many parameters can affect the results of the measurements, such as the working pH area, the temperature of the current reaction, the applicability of the assay to both hydrophilic and lipophilic compounds, and others [26,67,101,123]. Therefore, there is an imperative need to select the applicable assay in each case, as the ABTS and CUPRAC tests can detect both hydrophilic and lipophilic antioxidants and FRAP and FC exclusively detect hydrophilic antioxidants while others such as DPPH can only be applied to hydrophobic systems. At the same time, interferences that may appear could affect the color of the food matrix and can be absorbed in the same area as the antioxidants [97].

The TAC tests have the benefit of being able to quantitatively assess the antioxidant components of a sample. It takes a lot of time and effort to measure each antioxidant component separately [124]. Several studies on food, plants, and human body fluids have been the subject of several years of investigation. Cao et al. [110] and Prior et al. [60] observed no link between serum ORAC and TEAC or between serum FRAP and serum TEAC. Furthermore, to demonstrate how these approaches differ from one another, a comparative analysis of the antioxidant capacities of 30 plant extracts was performed using the DPPH, ABTS, and FRAP tests [125]. The FRAP and ABTS assays had the highest correlation (0.946) while the ABTS and DPPH assays had the lowest correlation (0.906).

Undoubtedly, one of the most common assays is the DPPH approach. The application of this test facilitates an understanding of a variety of chemical processes and offers several obvious advantages, such as affordability, experiment simplicity, reproducibility, applicability at room temperature, and automation possibilities [126]. However, the overlapping spectra of substances that are absorbed in the same wavelength range as DPPH is a significant drawback. For instance, anthocyanins exhibit significant absorption in the same wavelength range (500–550 nm) as DPPH, which could introduce interference into the data and affect how it is interpreted [57]. On the other hand, CUPRAC reagent is more stable and convenient to use than other chromogenic reagents (e.g., ABTS, DPPH). The CUPRAC assay works best at a pH of 7.0, which is very similar to the physiological pH (7.4) and simulates antioxidant action in natural settings. Furthermore, it is characterized by robustness in contrast to free radical reagents, such as DPPH, as it is not affected by physiological conditions such as air, humidity, and sunshine [26]. Additionally, CUBRAC, like the ABTS assay, is very selective because it has a lower redox potential than the Folin or ferric ion-based approaches. Additionally, the CUPRAC reagent does not cause oxidation of simple sugars or citric acid, which are not considered as real antioxidants, but the majority of phenolic antioxidants are readily oxidized due to their advantageous redox potentials. Moreover, the CUPRAC reagent can easily oxidize several antioxidants that are resistant to the FRAP, FOX, and FTC assays, with perfectly linear absorbance–concentration curves [97]. Although, this assay does not assess antioxidant enzymes or certain thiol antioxidants, such as glutathione [26,64].

Another favorable assay is the Folin–Ciocalteu test. Numerous benefits also exist for the use of FC to quantify TPC, including its ease of use, repeatability, and robustness. In fact, according to a previous report, there is a strong correlation in the Folin-determined concentration between FRAP and ABTS assays (0.946) in contrast with ABTS and DPPH assays (0.906) [17]. It does, however, have significant shortcomings. This test is sensitive to pH, temperature, and the reaction duration. Therefore, careful selection of the reaction state is crucial for achieving consistent and trustworthy findings. Due to the involvement of non-phenolic reducing agents present in the system when reducing the Folin–Ciocalteu reagent, TPC overestimation is a significant concern for the Folin–Ciocalteu test. Reducing sugars and certain amino acids are some of these pollutants [126].

Indirect measures, which are based on determining a sample’s capability to reduce a metal complex, can also be performed with FRAP. One major limitation of the FRAP assay is that an aqueous testing apparatus is required, but it provides quick, repeatable findings. Consequently, a water-soluble antioxidant must be used as the reference [23]. In addition, the propensity of blue to precipitate, form a suspension, and stain the measurement vat is a drawback of this FRAP test. Because of this, the timing of the addition of Fe^3+^ (FeCl_3_) is crucial and may lead to inaccuracies in the interpretation of the outcomes [26]. In fact, FRAP results can vary tremendously depending on the timescale of analysis. Moreover, after several hours of reaction time, the absorption of polyphenols such as caffeic acid, tannic acid, ferulic acid, ascorbic acid, and quercetin gradually increases. Some polyphenols have slower reactions and need more time to be detected while others react rapidly with iron complexes, leading to degradation into other compounds with differing or lower reactivity [60]. Therefore, a single-point absorption terminus might not be indicative of a finished reaction. Regarding its limitations, any substance that has a lower redox potential than the redox pair Fe^3+^/Fe^2+^ has the ability to reduce this system, raising the FRAP value and producing artificially high findings [60].

The FRAP assay has many similarities with the TEAC procedure, with the exception that TEAC is carried out at neutral pH and the FRAP assay is performed under acidic (pH 3.6) conditions [24]. The main advantage of TEAC is that it uses ABTS, which is soluble in both aqueous and organic solvent environments, allowing simultaneous assessment of hydrophilic and lipophilic antioxidants. Since the ABTS radical scavenging method can be tested over a wide pH range, it is useful for researching how pH affects antioxidant mechanisms in food-related components. Furthermore, this simplifies operations and allows for automated analysis. On the other hand, it could take a while to reach an endpoint due to the radical ABTS employed in the procedure, which does not reflect a physiological radical source. Although, due to the use of the synthetic ABTS radical cation, which is not present in food or biological systems, the TEAC assay has also been criticized for lacking biological relevance. As a result, numerous phenolic substances can interact with ABTS^•+^ because they have low redox potentials [26,97]. A previous study also suggests that there is no correlation between the HORAC and ORAC values [113]. ORAC measures the capability of absorbing peroxyl radicals while HORAC principally measures the ability to prevent metal-chelating radicals from doing so. Samples with high HORAC values are therefore anticipated to not necessarily have high ORAC values and vice versa. Due to the fact that many antioxidants are also metal chelators, the Fe(II)/H_2_O_2_ mixture suffers in a scavenging assay. This is also the reason why FC has replaced ORAC in many cases [78]. It is therefore impossible to determine whether the antioxidants are merely effective metal chelators or HO• scavengers. By converting Fe(III) to Fe(II), dietary antioxidants (such as vitamin C) can function as pro-oxidants and increase the rate of oxidation [24].

Finally, the TRAP, TBARS-TBA, and β-carotene bleaching assays are used for their applicability to many different carbonyl compounds formed from lipid peroxidation. Generally, there is a good correlation between the FOX and TBARS approaches. However, in the study conducted by DeLong et al. [72], the UV-induced increases were greater in TBARS plant tissues than in the FOX assay. In a previous survey by Bhuvaneswari et al. [127], FTC was used to evaluate the total phenolic content, flavonoid content, and antioxidant properties among different cultivars of *Piper betle L*. In comparison with other assays, no significant difference was found between ABTS and FOX while FOX was also as good as TBA and FRAP [127]. Moreover, the TRAP values for a given combination of antioxidant compounds are often lower than the TEAC values while the correlation between FRAP measurements is typically low [60,114]. Meanwhile, the TRAP and HORAC correlation coefficient is mentioned as 0.94 while in the case of ORAC-TRAP, the correlation is found to be r = 0.96 [114]. However, one major disadvantage of the TBA assay is that is not specific to MA, and this results in an overestimation of the MA concentration [101]. Additionally, due to the instability of the substrates utilized for lipid peroxidation, antioxidant assays based on the spectrophotometric methods of thiobarbituric acid-reactive substance production have low reproducibility. Finally, the bleaching β-carotene test is disadvantaged by its inability to be repeated, as the complexity of the reaction involving carotenes under oxygen shows antioxidant action at low oxygen concentrations and propagation of the oxidative chain in air-saturated solutions [128].

## 7. Conclusions

Natural and potent antioxidants are in demand for food and pharmaceutical products. Effective identification of sources of naturally occurring antioxidants and design of novel antioxidant compounds require reliable methods for antioxidant activity evaluation. Understanding the chemistry of the mechanisms, advantages, and limitations of the methods is critical for the proper selection of techniques for the valid assessment of antioxidant activity in specific samples or conditions. A number of chemical assays and food and biological model systems have been developed to determine the radical scavenging capacity, reducing power, and other specific attributes of antioxidants at the molecular or cellular level in addition to the overall oxidation inhibition in more complex food and biological systems. The antioxidant potential can be determined by various assays with specific mechanisms of action, including hydrogen atom transfer, single electron transfer, and targeted scavenging activities. These methods vary in terms of the antioxidant mechanism, substrate type, oxidation initiator, resulting expression, and ease of operation. The selection of an appropriate method or combination of assays is essential for valid assessment of antioxidant activity and eventually the potential of antioxidants as health-promoting agents or preservative food additives.

## Figures and Tables

**Table 1:** Reactive oxygen (ROS), nitrogen (RNS), and sulfur (RSS) species and their non-free-radical species.

Reactive Species			
	**ROS**	**RNS**	**RSS**
**Radical**	Peroxyl ROO•	Nitric oxide NO•	Alkoxyl-thiyl RS•
	Alkoxyl RO•	Nitrogen dioxide NO_2_•	Sulfide cation (RSR)•+
	Hydroxyl •OH		Disulfide anion (RSSR)•−
	Superoxide O_2_•^−^		Disulfide cation (R_2_S∴SR_2_)•+
	Bicarbonate HCO_3_•		Perthiyl RSS•
			Sulfinyl RSO•
			Sulfonyl RS(O)_2_•
			Sulfur trioxide anion SO_3_•^−^
			Sulfate anion SO_4_•^−^
**Non-Radical**	Hydrogen peroxide	Peroxynitrite ONOO^−^	Sulfate SO_4_^2−^
	H_2_O_2_	Nitrosyl cation NO^+^	Dithionate S_2_O_6_^2−^
	Singlet oxygen ^1^O_2_	Nitrous acid HNO_2_	Sulfite SO_3_^2−^
	Ozone O_3_	Nitryl chloride NO_2_CL	Disulfide R_2_S
	Peroxide R_2_O_2_	Nitroxyl anion NO^−^	Hydrogen sulphide H_2_S
	Alcohol ROH	Dinitrogen trioxide N_2_O_3_	Disulfide-S-dioxide RS(O_2_) SR
	Organic peroxide ROOH	Dinitrogen tetraoxide N_2_O_4_	Disulfide-S-monoxide RS(O)SR
		Peroxynitrous acid ONOOH	Sulfenic acid RSOH
			Thiol RSR
			Tetrathionate S_4_O_6_^2−^
			Peroxodisulfate S_2_O_8_^2−^

**Table 2:** Spectrophotometric parameters of each assay.

Assay	nm	Principle of Method	Determination	Color Shifting		Reference
				From	To	
DPP	515–520	Antioxidant reaction with free organic radicals	Colorimetry	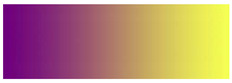		[10,59,61]
Folin–Ciocalteu	760–765	The reductive capacity of antioxidants to determine the total phenolic content	Colorimetry	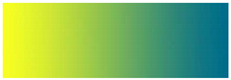		[59,62,63]
CUPRAC	450–490	Measures TAC of the reduction of Cu (II) to Cu (I) by antioxidants	Colorimetry	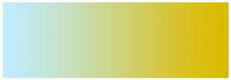		[26,64,65]
FRAP	593	Measures the antioxidant potential through the reduction of Fe (III) to Fe (II) by antioxidants	Colorimetry	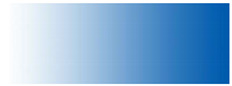		[23,26,46,65]
ABTS	414, 645–650, 734, 815–820	Measures the relative ability of antioxidants to scavenge the ABTS generated in the aqueous phase	Colorimetry	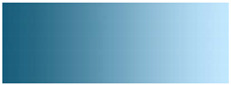		[6,23,64,65,66,67]
ORAC and HORAC	485–525 and 485–535	Antioxidant reaction with peroxyl radicals and quench OH radicals generated by a Co(II)-based Fenton-like system	Loss of fluorescence of fluorescein	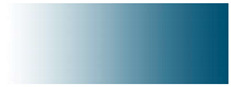		[5,24,26,66,68]
TBA-TBARS	532–535	Based on the reactivity of malondialdehyde (MDA) with TBA to produce a red color	Colorimetry	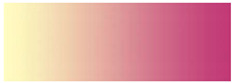		[23,69,70,71]
FOX	550–560	Measure the levels of hydrogen peroxide in biological systems by the oxidation of Fe(II) to Fe(III)	Colorimetry	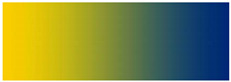		[23,72,73]
FTC	500	Measure the levels of hydrogen peroxide as the ferric ion is converted by an oxidant from a ferrous ion	Colorimetry	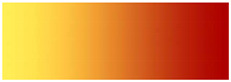		[23,71,74]
β-Carotene Bleaching Assay	440	Measure the levels of peroxyl radicals as β-carotene blenched	Colorimetry	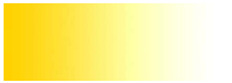		[23,75]
Hydrogen peroxide scavenging	460	Total oxidant scavenging capacity of antioxidants	Fluorescence	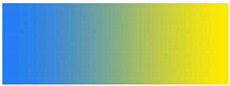		[10,76]
Superoxide radical scavenging	560–562	Total oxidant scavenging capacity of antioxidants	Colorimetry	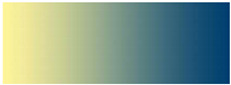		[76,77]
Nitric oxide radical scavenging	540	Total oxidant scavenging capacity of antioxidants	Colorimetry	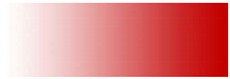		[76]
Peroxynitrite Scavenging	485, 505, 529–530, 611	Total oxidant scavenging capacity of antioxidants	Fluorescence	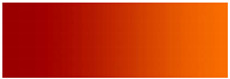		[78,79]

## Data Availability

The data presented in this study are available in this article.
